# Older adults’ perspectives on big data use in hip fracture research: a qualitative study

**DOI:** 10.1186/s12877-026-07618-0

**Published:** 2026-05-11

**Authors:** Louis J. Koizia, Su-Lee Xiao, Benjamin H. L. Harris, Michael B. Fertleman

**Affiliations:** 1 Cutrale Perioperative and Ageing Group, Department of Bioengineering, Imperial College London, London, UK; 2 Imperial College Healthcare NHS Trust, London, UK; 3 St. Catherine’s College, University of Oxford, Oxford, UK; 4 Department of Oncology, University of Oxford, Oxford, UK; 5 Institute for Liver and Digestive Health, Division of Medicine, University College London, London, UK; 6 Department of Psychosocial Rehabilitation, Medical University of Lodz, Lodz, Poland; 7 Collaborating Centre for Values-based Practice, St Catherine’s College, Manor Rd, Oxford, UK

**Keywords:** Older adults, Hip fracture, Big data, Qualitative research, Thematic analysis

## Abstract

**Background:**

Big data approaches are increasingly applied to hip fracture research, linking clinical, imaging, environmental and genomic data. Yet the views of older adults, whose data underpin these efforts, remain under-explored.

**Methods:**

Focused, semi-structured interviews were conducted with older adults recruited from an orthopaedic ward at an inner city teaching hospital. Eligibility included age 60 years or older and a hip fracture within the previous three months. Fifteen participants took part. Interviews were audio recorded at the bedside, transcribed verbatim, anonymised and analysed using Braun and Clarke’s reflexive thematic analysis.

**Results:**

Participants commonly reported limited familiarity with the term big data yet expressed broad support for its use in healthcare. Five themes described their perspectives: limited understanding alongside support for healthcare use; consent and trust as the moral foundation for sharing; altruism and social benefit; preference for treatment and recovery over prevention; and the importance of communication, comprehension and inclusion, including clear feedback and recognition of the digital divide. Hospitals and universities were the most trusted custodians. Governments and private companies elicited scepticism. Many viewed fractures as inevitable, which dampened enthusiasm for preventive analytics, while tangible improvements to treatment and coordination were strongly valued.

**Conclusion:**

There is clear acceptance of big data use amongst older adults with many perceived benefits. They prioritise trust, clear consent processes, visible public benefit and opportunities for feedback. Framing data initiatives around improving patient recovery and coordination of care, as well as communicating how fracture prevention supports independence, may strengthen social license. Ethical stewardship, transparency and co-design with older adults are essential to sustain confidence and ensure data-driven innovation delivers meaningful benefit.

**Supplementary Information:**

The online version contains supplementary material available at 10.1186/s12877-026-07618-0.

## Introduction

Hip fractures are among the most serious and costly injuries in later life, leading to substantial disability, loss of independence and excess mortality [1, 2]. Big data approaches now allow the integration of diverse intrinsic and extrinsic risk factors and support large-scale modelling to inform more timely and tailored strategies in hip fracture care. Recent genomic and population-level analyses demonstrate how large, multidimensional datasets can generate new insights into the aetiology and risk factors for hip fractures [3, 4], as well as other complex diseases [5–7]. Electronic health record based models have also shown the ability to predict short-term falls risk using machine learning algorithms that combine diagnoses, medications, clinical characteristics and laboratory data, with emerging potential to incorporate environmental and genomic variables to improve accuracy [8]. However, translating such advances into preventive or clinical action depends not only on technical capability but also increasingly on social legitimacy and public trust. Public perceptions of emerging health technologies vary considerably across populations, and older adults’ willingness to share health and personal data is shaped by trust, perceived benefit and privacy concerns, highlighting the importance of inclusive engagement when designing data-driven research initiatives [9, 10]. International frameworks, including the World Health Organisation (WHO) Decade of Healthy Ageing and the Organisation for Economic Co-operation and Development (OECD) Recommendation on Health Data Governance, emphasise fairness, inclusivity and transparency in the use of health data for research and innovation [11]. Despite this, few studies have examined how older adults themselves perceive the use of large-scale health and environmental data for falls and hip fracture research.

Older adults’ experiences with digital technologies are heterogeneous [12]. While many engage with some form of digital health information, digital exclusion related to usability barriers and broader social determinants of the digital divide persists, and is associated with greater functional dependence and poorer health outcomes [13–15]. Big data systems may reinforce inequalities if datasets are incomplete or overlook under-represented groups, like older adults. Engaging older adults in the governance and co-design of health data initiatives is therefore important for ethical, trustworthy and sustainable implementation. Recent research examining older adults’ online health information behaviour has highlighted the importance of trust, perceived credibility and social motivations in shaping how health information is shared and interpreted. However, there remains limited research exploring how older adults perceive the use of large-scale health data in research and clinical care [16]. The aim of this study was to explore older adults’ perceptions of big data use in hip fracture research, and to understand the social and ethical factors that influence data sharing and participation.

## Methods

### Design and setting

This exploratory qualitative study used focused, semi-structured interviews conducted at St Mary’s Hospital, Paddington, London (Imperial College Healthcare NHS Trust).

### Participants and sampling

Older adults were eligible if they were aged 60 years or older, had sustained a hip fracture within the preceding three months, and had capacity to provide informed consent. Participants were excluded if they were unable to communicate in English or if clinical staff advised that participation would be inappropriate due to medical instability. Purposive sampling sought variation in age, gender, education, and digital literacy. Fifteen participants were recruited from orthopaedic wards. Three eligible patients declined to participate due to clashes with multidisciplinary team commitments (*n* = 2) or fatigue (*n* = 1). Capacity to provide informed consent was assessed by the clinical team in accordance with standard ward practice prior to recruitment.

### Data collection

The project was registered as a Quality Improvement Project within Imperial College Healthcare NHS Trust. Written informed consent was obtained before each interview. Interviews were conducted face-to-face at the bedside during participants’ inpatient recovery period, lasting approximately 30–45 min. Interviews were conducted by Louis J. Koizia (consultant geriatrician) and Su-Lee Xiao (specialist registrar in geriatrics), both members of the orthogeriatric team involved in the clinical care of patients with hip fracture. They were not part of the orthopaedic surgical team responsible for operative management. Efforts were made to maintain a conversational, non-clinical interview environment, emphasising participants’ experiences and perspectives. Bed curtains were drawn to maintain privacy and interruptions from clinical care activities were minimised. The semi-structured interview guide explored participants’ understanding of big data and its relevance to healthcare, attitudes toward the collection and use of health data for research, and perceived benefits and risks of using large datasets for prevention and care (Supplementary Methods). At the start of each interview, participants were provided with a brief explanation of big data in healthcare to establish a shared conceptual starting point. Discussion nevertheless often extended beyond big data in a narrow technical sense, with some participants relating the topic to broader experiences of research participation, data collection and health information use.

Interviews were audio-recorded, transcribed verbatim and anonymised during transcription. Field notes documented context and non-verbal observations.

### Analysis

The study adopted an exploratory qualitative design and did not employ a predefined theoretical framework. Instead, an inductive approach guided by Braun and Clarke’s reflexive thematic analysis was used [17]. Reflexive thematic analysis was chosen because it allows flexible exploration of participants’ experiences and meanings without imposing predefined theoretical assumptions. Initial open coding was conducted line by line, with codes iteratively organised into preliminary categories and interpretively developed into overarching themes. Two researchers independently coded all transcripts, with discrepancies resolved through discussion and consensus. The researchers engaged in iterative discussion throughout the analytical process to reflect on interpretations and refine themes. NVivo 14 facilitated systematic data organisation and supported the iterative refinement of themes. Participant checking (member checking) was not undertaken. Analytical rigour was supported through independent coding and iterative discussion during theme development.

### Ethical approval

As a registered Quality Improvement Project, formal NHS Research Ethics Committee review was not required. All participants provided written informed consent. Data were anonymised and handled securely in accordance with institutional governance procedures.

## Results

### Participant characteristics

Fifteen participants took part: nine women and six men, aged 70 to 91 years (Table 1). Educational backgrounds ranged from secondary school to postgraduate qualifications. Former occupations included teaching, journalism, engineering, management, retail, clerical work and social work. Digital literacy varied from basic mobile phone use to confident daily use of computers, smartphones and tablets. Most participants were retired, although some remained active in voluntary or community roles.

**Table 1:** Overview of participant demographic and contextual characteristics

Age (yrs)	Gender	Living situation	Highest level of education	Former / main occupation	Previous healthcare experience	Digital literacy
80	F	Married; lives with husband	University	Executive assistant; regional merchandiser	Inpatient - open-heart surgery	Moderate - computer + smartphone
83	F	Married; lives with husband	Secondary	Retired teacher	Multiple hospital stays	Moderate - smartphone & tablet
72	F	Lives with husband	University	HR director; management consultant	GP visits; inpatient (current)	High - smartphone, tablet, computer
79	F	Lives alone (widowed)	Secondary	Coffee-shop worker	Hospital admission; GP for asthma	Low - smartphone only
87	F	Lives alone; partner in care home	Left school at 15	Photographic retoucher / colourist	GP and hospital care	Moderate - smartphone + laptop
81	M	Lives alone (widower)	University	Documentary film-maker	GP and hospital care	Low - laptop only, limited use
70	M	Married; lives with wife	University	Pharmacy manager	GP + hospital	High - smartphone, computer
75	F	Lives with spouse	Secondary	Factory secretary	GP + hospital	Low - smartphone only
82	M	Married; lives with wife	University	Engineer	Multiple hospital admissions	High - smartphone + PC
91	F	Lives alone	Secondary	Social worker	Hospital admission for fracture	Low - basic mobile phone
73	M	Married	Secondary	Accountant	GP + hospital	Moderate - smartphone + tablet
82	F	Widowed; lives with daughter	Secondary	Factory secretary	GP + hospital	Low - smartphone
84	M	Married	University	Retired journalist	Hospital stay for fracture	High - smartphone + laptop
76	F	Lives with husband	Secondary	Shop assistant (retail)	GP visits	Low - smartphone only
89	M	Lives alone	Secondary	Clerical officer	GP + rehabilitation	Low - basic mobile phone

Participants represented diverse educational, occupational and digital literacy backgrounds, providing a range of perspectives on healthcare data use (*n* = 15)

### Theme 1: limited familiarity with big data alongside broad support for healthcare use

Most participants were unfamiliar with the term big data and described little or no prior understanding of it. One participant stated, *“I don’t know.” (Participant 4*,* 88-year-old female*), while another reflected, *“I know data. But big*,* that is a new phrase to be added to big data…” (Participant 6*,* 73-year-old male)*. Despite limited technical understanding, most expressed strong support for the use of big data in healthcare. It was viewed as part of modern medicine, associated with faster decision making and improved care. A minority questioned its relevance to fractures, which they framed as accidents or consequences of ageing. Support was driven primarily by trust in scientific progress and institutional goodwill, rather than by detailed understanding of big data. This suggests that acceptance of data-driven healthcare may depend less on technical understanding and more on broader trust in medical progress and institutions. Limited familiarity with the term also meant that some participants appeared to interpret big data through the more familiar lens of research participation and health data collection more generally.

### Theme 2: consent and trust as moral foundations for sharing

Consent and trust were repeatedly brought up as the ethical basis for data sharing. Seeking permission was experienced as a sign of respect and accountability. Hospitals and universities were consistently trusted as data custodians, whereas governments and private companies elicited scepticism due to perceived political or commercial motives. Anonymisation reassured some participants. Clear explanations about purpose, access and safeguards were considered a basic right and a condition for ongoing confidence. Together, these accounts indicate that trust and consent function not merely as procedural requirements but as moral signals of respect and accountability within data governance. Illustrative comments included:

*“It is appropriate to ask permission of people.” (Participant 1*,* 80-year-old female)*.

*“Hospitals and universities*,* yes. Government or companies*,* I am not so sure.” (Participant 4*,* 88-year-old female)*.

*“I think they should be asked each time. Shouldn’t just do it*,* you should be asked.” (Participant 11*,* 72-year-old female)*.

### Theme 3: Altruism and social benefit

Altruistic motivations strongly shaped attitudes toward data use. Sharing data was described as an act of civic contribution, helping “future generations” or “other families who go through this”. Many participants referenced personal or family engagement with research and saw participation as morally worthwhile when it advanced the public good. Practical challenges such as fatigue, pain or transport occasionally limited involvement, but these were framed as logistical barriers, not reluctance in principle. These narratives position participation in data sharing as a form of civic contribution, where personal information is viewed as a resource that can generate collective social benefit. Illustrative comments included:

*“If I felt any information I had was going to be useful to others*,* of course I would help.” (Participant 8*,* 75-year-old female)*.

*“Anything for the good cause for the future*,* I’m quite happy.” (Participant 2*,* 70-year-old male)*.

*“I’m certainly agreeable to it because of the advantages to help other people for the future. I think a lot of progress is being made daily. And that helps us all.” (Participant 1*,* 80-year-old male)*.

### Theme 4: Preference for treatment and recovery over prevention

Participants valued big data use most when it produced visible improvements in treatment, recovery and coordination of care. Many perceived fractures as largely inevitable, a belief that dampened enthusiasm for preventive analytics or complex risk prediction models. When prevention was supported, it was conceptualised pragmatically. Immediate, tangible benefits were prioritised over abstract predictions about future risk. This framing reflects a pragmatic orientation toward healthcare, where research is valued most when it produces visible improvements in treatment and recovery rather than abstract future risk prediction. Illustrative comments included:

*" I don’t see how you can prevent a hip fracture if you have a trip or a fall.” (Participant 10*,* 83-year-old female)*.

*“Anybody could slip. I think my sort of accident was purely a fall*,* wasn’t it? A very*,* very unexpected fall.” (Participant 8*,* 75-year-old female)*.

*“Preventing is difficult to think about really*,* because as people get older they fall.” (Participant 7*,* 82-year-old male)*.

### Theme 5: Communication, comprehension and inclusion

Clear, jargon-free communication emerged as a pervasive need. Several participants found data-related terminology confusing and emphasised the importance of simple explanations, opportunities to ask questions and feedback about how their information was used. The digital divide was evident: some lacked internet access, confidence with technology or trust in online systems. Participants emphasised the importance of choice, plain-language consent and accessible methods of feedback. Inclusion, partnership and transparent communication were viewed as essential to sustaining support for big data initiatives. These accounts highlight that communication and accessibility are central to meaningful participation in data-driven research, particularly for populations who may feel excluded by technical language or digital barriers. Illustrative quotations included:

*“I’ll be quite honest*,* this age*,* my age group*,* everything is beyond us. It truly is a different language. Better explanations would help*,* and simplify everything.” (Participant 14*,* 87-year-old female)*.

*“Because in terms of the care*,* it’s not just the surgical intervention and recovery*,* it’s also about how patients are communicated with and involved in care plans.” (Participant 12*,* 72-year-old female)*.

*“Giving them some kind of outcome information… so they say*,* ‘Well*,* this is what on the whole we’ve found with the cases.’” (Participant 4*,* 88-year-old female)*.

## Discussion

**Fig. 1:**
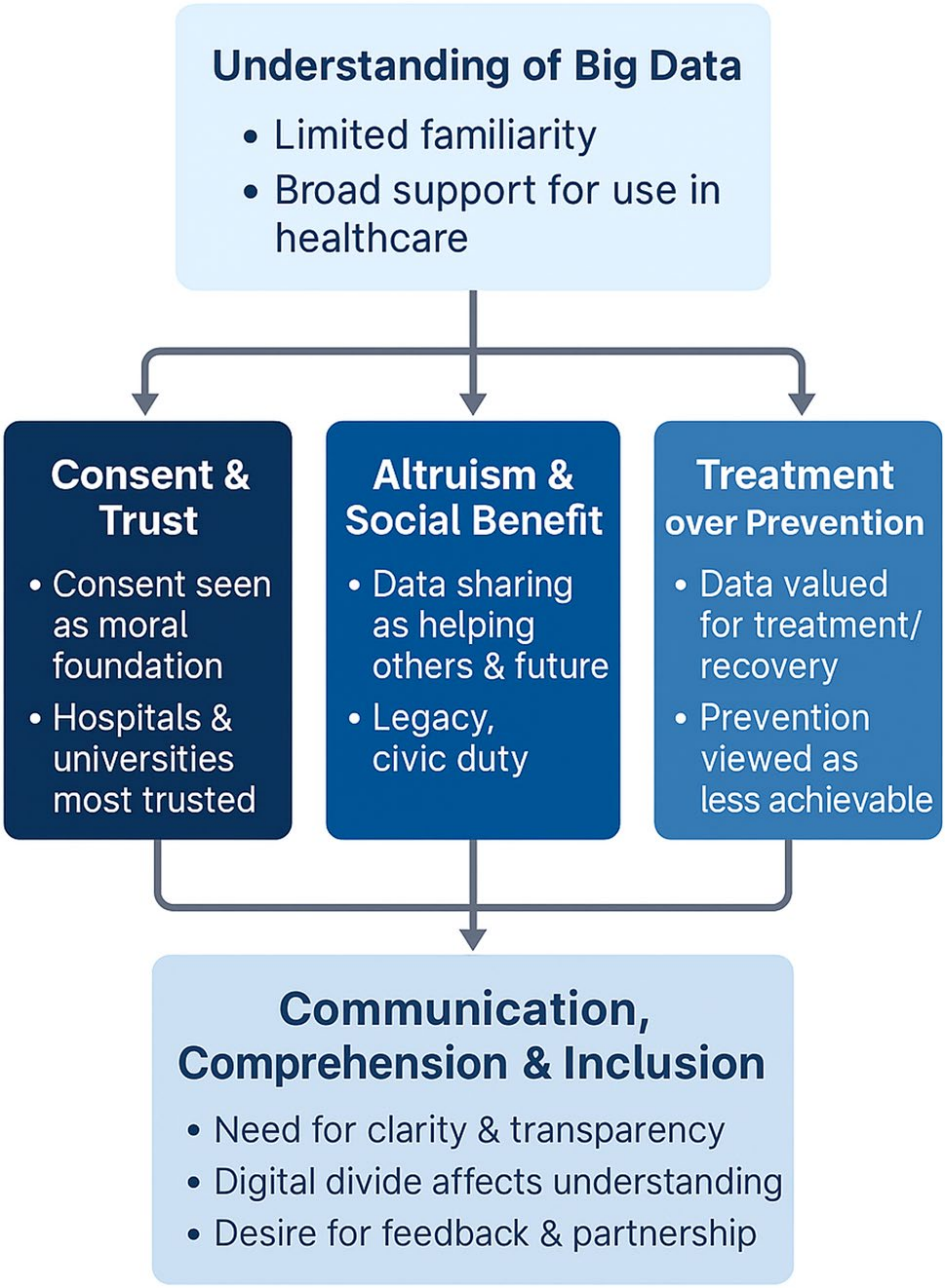
Emergent themes on older adults’ perspectives toward big data in hip fracture research. Five interconnected themes illustrated participants’ attitudes to data use in healthcare (*n* = 15). Limited understanding coexisted with broad support. Consent and trust underpinned sharing, motivated by altruism. Participants valued treatment improvements over prevention and emphasised clear communication and inclusion

This study suggests that older adults’ acceptance of big data use in hip fracture research is shaped by altruism, trust and perceived social benefit, rather than by technical understanding (Fig. 1). Consent and trust formed the moral foundations for participation, consistent with broader emerging literature emphasising the importance of transparency and fair governance in health data use [18–20].

Hospitals and universities were regarded as the most trusted institutions, whereas governments and private companies were approached with caution due to perceived political or commercial motives. This aligns with previous findings that hospitals are often viewed as places of safety following hip fracture [21]. Participants’ concerns mirrored wider public responses to high-profile data incidents, such as the failure of the NHS Care.data programme, a national initiative intended to link primary care and hospital records for research that was halted following public concerns about data governance and the 2017 WannaCry ransomware cyberattack that disrupted NHS hospital systems across the United Kingdom [22, 23]. These breaches of security eroded confidence in institutional data stewardship and heightened fears about surveillance, misuse and loss of control.

Participants were primarily motivated by altruism and a strong desire to contribute to the greater social good, often framing their involvement as a way to help future patients and improve healthcare systems. However, they placed the greatest value on research applications that offered tangible benefits for treatment and recovery, particularly those that are modifiable and could shorten hospital stays, reduce complications, and restore independence. Participants appeared most supportive of research that translated directly into practical improvements in care pathways. Similar translational approaches in other areas of medicine have demonstrated how evidence can be operationalised through structured clinical protocols and standard operating procedures to improve outcomes [24].

Many participants perceived hip fractures as largely unavoidable, attributing them to ageing and frailty rather than modifiable risk factors. Consequently, preventive analytics and large data-linkage studies, such as the REDUCE study, which examines organisational factors associated with hip fracture outcomes [25, 26], may appear more abstract and less personally meaningful unless their findings are clearly connected to tangible improvements in care. In contrast, platforms like the World Hip Trauma Evaluation (WHiTE), which clearly links modifiable treatments to patient outcomes appear more immediately relevant and appealing [27]. This highlights the importance of narrative and framing preventive interventions and linkage studies in ways that resonate with patients’ priorities, emphasising practical and modifiable benefits such as fall prevention, mobility support, and maintaining independence at home.

These findings contribute important social and ethical insight into ongoing data-driven work in hip fracture research. While big data approaches have demonstrated value in genomics, risk stratification and predictive modelling, our results highlight that older adults evaluate such initiatives through the lens of trust, purpose and institutional integrity. This aligns with international health data governance guidance emphasising fairness, inclusivity and transparency.

An important finding was that participants did not always distinguish sharply between big data, research data collection and broader health information use. This likely reflects the limited familiarity with the term “big data” observed in Theme 1 and suggests that older adults often interpret data-driven research through more familiar experiences of consent, research participation and information sharing. Rather than undermining the findings, this overlap helps explain why issues such as communication, trust and participation were so prominent across themes.

### Implications for practice, research, and policy

This work reinforces several practical and policy implications for future big data projects. Participants emphasised the importance of clear explanations and transparency when discussing health data and research participation. Supportive communication during consent processes may therefore help older adults feel more comfortable participating in research involving large health datasets. These considerations involve both clinicians and wider research governance structures responsible for consent procedures. Participants also frequently highlighted the importance of opportunities to ask questions and receive understandable information about health data use, suggesting that improving communication and patient–family engagement may benefit broader inpatient care settings, particularly when introducing complex or unfamiliar concepts related to data use in research. These findings also reinforce the importance of patient-centred approaches to data-driven healthcare and research, emphasising communication, shared understanding, meaningful patient engagement, and situational judgement when introducing complex health technologies and research concepts [31, 32, 33].

Participants’ accounts also highlighted aspects of the recovery experience following hip fracture that have implications for research participation and care. Some described fatigue and fluctuating clarity during recovery, suggesting that qualitative methodologies may need to accommodate these circumstances through approaches such as pacing interviews, shorter or repeated conversations, and careful capacity assessment to ensure participation remains ethical. Participants’ experiences further underscored the emotional and psychological dimensions of recovery, including vulnerability and uncertainty during hospitalisation. These insights reinforce the importance of relational care, continuity, and psychological support alongside physical rehabilitation.

### Strengths and limitations

Strengths of the study include focused bedside interviews soon after hip fracture, heterogeneity in demographics and rigorous inductive reflexive thematic analysis. Limitations of the study are that it was carried out at a single site, the interviews were conducted only in English, and there is a possibility that recovery-related stress influenced responses. Conducting interviews at the bedside may have introduced environmental distractions associated with ward settings; however, interviews were conducted at times convenient for participants and efforts were made to maintain privacy and minimise interruptions. Further, social desirability bias cannot be excluded. Patients with significant cognitive impairment who were unable to provide informed consent were excluded from the study. Some participants described experiencing poorer cognitive function and distress following their fracture, phenomena that are well documented in the literature [28, 29]. These factors may have influenced participants’ responses, although all participants appeared to have capacity at the time of interview.

Future research could explore a wider range of institutional and patient contexts through multi-site recruitment, which may provide additional insight into how perceptions of health data use vary across healthcare settings. Including community-dwelling older adults without recent fractures may also help reduce bias associated with the acute recovery period. Employing multilingual approaches and mixed-method designs, such as combining focus groups with structured questionnaires, may enable exploration of collective reasoning and shared perspectives around health data use. Repeated interviews at different stages of recovery, alongside triangulation of accounts and involvement of families or carers, may also help capture how attitudes toward research participation and data sharing evolve over time.

Our findings remind us that big data initiatives are not merely technical undertakings reliant on *data analysis and content knowledge* [30]; they are fundamentally relational. Older adults want to see purpose, integrity and feedback in how their data are used. Designing systems that honour these expectations will be crucial to ensuring that data-driven approaches support, not distance, the people at the centre of hip fracture care.

## Conclusion

Overall, this study contributes to a growing body of geriatric qualitative research emphasising the importance of listening to older adults’ lived experiences. It highlights the nuanced ways in which older people balance vulnerability with altruism and resilience, and illustrates that their perspectives must continue to meaningfully shape both clinical practice and research ethics.

## Supplementary Information

Supplementary Material 1.

## Data Availability

The datasets generated during the current study are not publicly available due to institutional governance. Data are available from the corresponding author on reasonable request, subject to appropriate approvals.

## Abbreviations

WHO: World Health Organization
OECD: Organisation for Economic Co-operation and Development
WHiTE: World Hip Trauma Evaluation
