# Supplementary material for: Older adults’ perspectives on big data use in hip fracture research: a qualitative study
Source: BMC Geriatr. 2026 May 11;26:903. doi: 10.1186/s12877-026-07618-0 (PMC13335330; doi:10.1186/s12877-026-07618-0)
Supplement: Supplementary file 1 — Supplementary Material 1. [file 12877_2026_7618_MOESM1_ESM.docx]

**Supplementary methods:** Semi-structured interview guide

**Demographic Data:**

- Age
- Gender
- Marital status / household composition
- Educational background
- Professional life (past occupation/s)
- Previous experience with healthcare services (hospital, primary care, rehabilitation)
- Previous experience with digital tools (e.g. smartphone, online portals, wearables)

–

**Understanding of Big Data**

1. What does the term *Big Data* mean to you?
2. Have you heard about it being used in healthcare before? If so, in what context?
3. Do you think hip fractures are a health problem where the use of Big Data could make a difference?

**Attitudes Toward Use in Healthcare**

1. How do you feel about your health information being collected and used as part of Big Data for research on hip fractures?
2. What benefits do you think the use of Big Data could bring for preventing or treating hip fractures?

**Privacy, Consent, and Trust**

1. How important is it to you that your consent/permission is obtained before your health information is used?
2. Who would you trust more with your health information — hospitals, universities, government, or private companies?
3. Who would you trust the most with your information?
   1. Hospital
   2. Universities
   3. Government
   4. Private Companies
4. Would you feel differently if your health information were anonymised compared to not?

**Perceived Benefits and Risks**

1. How do you think Big Data could be used to reduce the chance of older people breaking their hip?
2. What risks or downsides do you see in using hip fracture data?

**Participation and Involvement**

1. Would you like to be involved in decisions about how fracture data are used?
2. If invited to take part in a study on hip fractures using big data, what would make you say yes or no?

**Ethical Considerations**

1. Do you think it’s ethical to use your fracture data without asking each patient individually?
2. Should researchers be allowed to link fracture data with other health information (like GP records or scans) without asking every time?
   Should companies that profit from fracture treatments have the same access to data as universities and hospitals?

**Suggestions for Improvement**

1. What could be done to help older adults feel more comfortable about their fracture data being used in research?
2. What advice would you give researchers trying to use Big Data to improve fracture care and prevention?
